# Caveolin-1 regulates lung cancer stem-like cell induction and p53 inactivation in carbon nanotube-driven tumorigenesis

**DOI:** 10.18632/oncotarget.1956

**Published:** 2014-05-08

**Authors:** Sudjit Luanpitpong, Liying Wang, Todd A. Stueckle, William Tse, Yi Charlie Chen, Yon Rojanasakul

**Affiliations:** ^1^ Pharmaceutical and Pharmacological Sciences Program, West Virginia University, WV 26506, USA; ^2^ Mary Babb Randolph Cancer Center, West Virginia University, WV 26506, USA; ^3^ Pathology and Physiology Research Branch, Health Effects Laboratory Division, National Institute for Occupational Safety and Health, Morgantown, WV 26505, USA; ^4^ College of Science, Technology and Mathematics, Alderson Broaddus University, Philippi, WV 26416, USA

**Keywords:** caveolin-1, p53, lung, cancer stem-like cells, tumorigenesis, carbon nanotubes.

## Abstract

Cancer stem cells (CSCs) may represent targets for carcinogenic initiation by chemical and environmental agents. Recent studies have raised a concern over the potential carcinogenicity of carbon nanotubes (CNTs), one of the most commonly used engineered nanomaterials with asbestos-like properties. Here, we show that chronic (6-month) exposure of human lung epithelial cells to single-walled (SW) CNTs at the workplace-relevant concentration induced an emergence of lung CSCs, as indicated by the induction of CSC tumor spheres and side population (SP). These CSCs, which were found to overexpress tumor promoter caveolin-1 (Cav-1), displayed aggressive cancer phenotypes of apoptosis resistance and enhanced cell invasion and migration compared with their non-CSC counterpart. Using gene manipulation strategies, we reveal for the first time that Cav-1 plays an essential role in CSC regulation and aggressiveness of SWCNT-transformed cells partly through p53 dysregulation, consistent with their suggested role by microarray and gene ontology analysis. Cav-1 not only promoted tumorigenesis in a xenograft mouse model but also metastasis of the transformed cells to neighboring tissues. Since CSCs are crucial to the initiation and early development of carcinogenesis, our findings on CSC induction by SWCNTs and Cav-1 could aid in the early detection and risk assessment of the disease.

## INTRODUCTION

Lung cancer is the leading cause of cancer-related death, and environmental and occupational exposure is a major cause of most cases [1,2]. Evolving research in stem cells and cancer biology have provided strong evidence for the existence of cancer stem cells (CSCs) in various human solid tumors, including brain, breast, bone marrow, prostrate, colon, and lung [3,4]. These CSCs are potential driving force of tumor initiation and progression due to their self-renewal and tumorigenic properties [5,6]. Induction of CSCs from non-tumorigenic cells may initiate carcinogenesis. While carcinogenesis induced by various chemicals and environmental agents including cigarette smoke, air pollution, and heavy metals has been extensively studied [7-9], relatively little has been carried out or known about the cancer risk caused by nanomaterial exposure, notably carbon nanotubes (CNTs) which share similar properties with asbestos fibers, a known human carcinogen [10-13]. Identifying CSC induction and its regulation by CNTs might lead to a better understanding of CNT carcinogenesis.

Typical developmental period for fiber-induced lung cancer in humans is 30-40 years [14]. The present study was undertaken to investigate whether chronic exposure of human lung epithelial cells to CNTs can induce CSCs, and if so by what mechanisms. Recently, a number of proteins have been identified to have a high prognostic correlation with lung cancer. Among these, a plasma membrane-associated protein caveolin-1 (Cav-1) has gained a considerable attention, since its expression has been linked to the advanced stages of squamous and non-small cell lung cancers [15-17]. Poor prognosis and reduced disease-free and overall survival were observed in Cav-1-positive patients [16]. Experimentally, an up-regulation of Cav-1 was shown to potentiate various steps in tumor progression, metastasis and chemoresistance [18,19], anchorage-independent growth (anoikis resistance) [20,21], and cell migration and invasion [22,23].

Here, we demonstrate that single-walled (SW) CNTs can trigger lung epithelial cells to initiate CSCs, which exhibit aggressive cancer behaviors of apoptosis resistance and enhanced cell motility. These CNT-derived CSCs overexpressed Cav-1 which was investigated in this study for its role in CSC regulation and tumorigenesis using gene manipulation and murine xenograft strategies. Our results indicate a novel role of Cav-1-p53 axis in CSC regulation, which could be important in lung tumorigenesis and metastasis.

## RESULTS

### Chronic SWCNT exposure induces CSCs

**Figure 1:**
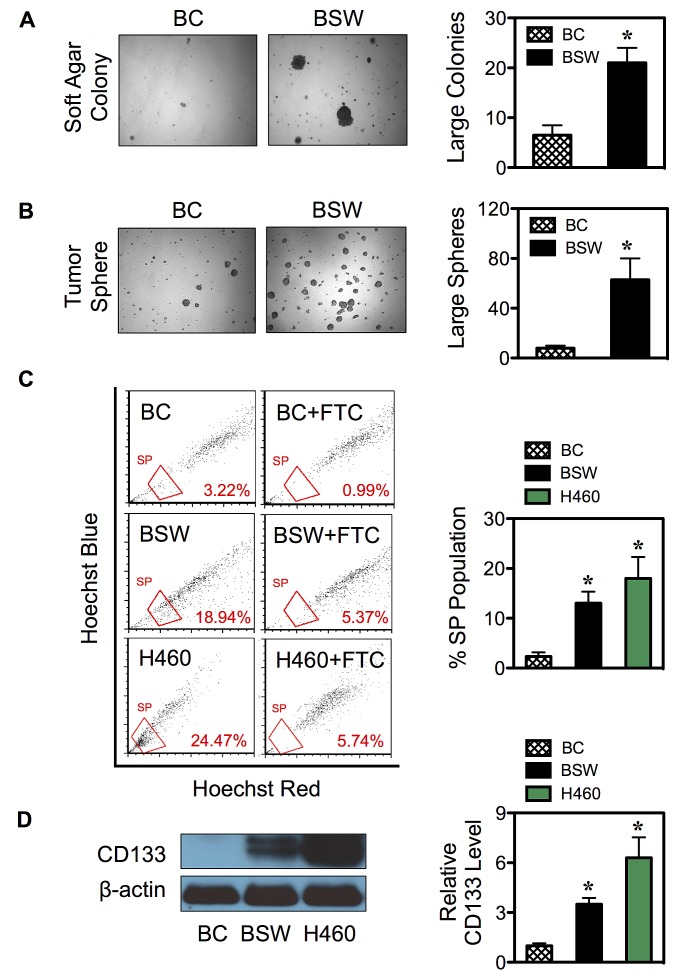
SWCNTs induce CSCs from human lung epithelial cells (A, B) Non-tumorigenic lung epithelial BEAS-2B cells were continuously exposed to SWCNTs for 6 months, then cultured in SWCNT-free medium for more than 20 passages, and designated as BSW cells. After which they were analyzed for soft agar colony formation (A) and tumor sphere formation (B). Passage-matched control BC cells were grown in the same background level of dispersant. (C) Analysis of side population (SP) in BC, BSW, and H460 lung cancer cells in the presence or absence of fumitremorgin c (FTC) using FACS. SP cells (*box*) were determined by their disappearance in the presence of FTC and were plotted as a percentage of pool population. (D) CD133 protein expression in BC, BSW, and H460 cells was determined by Western blotting. β-actin was used as a loading control. Data are mean ± SD (n = 3 or 4). *p < 0.05 ***vs.*** control BC cells.

We have previously shown that chronic exposure of human lung epithelial cells to non-cytotoxic concentration of SWCNTs induced malignant transformation as assessed by soft-agar colony formation, a common measure of anchorage-independent growth, and mouse xenograft tumorigenesis assays [12]. However, the fundamental understanding and regulatory mechanisms of tumorigenesis are not well understood. In this study, we first showed that SWCNT-induced malignant transformation was irreversible, since cells that were propagated for more than 20 passages in the absence of SWCNTs still maintained the ability to grow on soft agar (Figure 1A). We next tested whether chronic SWCNT exposure can induce CSCs, a subpopulation that may drive tumor initiation and progression. Fundamental properties of CSCs are their ability to self-renew and generate differentiated progeny [24,25]. Tumor sphere assay under non-attached and serum-starved conditions was used to determine the CSC self-renewal capability. Figure 1B shows that while passage-control BC cells minimally survived, chronic SWCNT-exposed BSW cells formed large floating spherical colonies. These results suggest the existence of cells that possess the characteristics of CSCs and their acquisition from non-tumorigenic lung epithelial cells by SWCNTs. The BSW cells that were passaged from first-generation tumor spheres preserved the ability to form second-generation spheres (not shown), thus confirming their renewal and repopulation capability.

The ability to efflux Hoechst dye via the multidrug resistance transporter ATP-binding cassette sub-family G member 2 (ABCG2) as identified by side population (SP) has proven to be a valuable marker for CSCs from various solid tumors and cancer cell lines [4,24,26]. Chronic SWCNT-exposed BSW cells and passage-control BC cells were stained with Hoechst 33342 in the presence or absence of fumitremorgin C (FTC), a specific inhibitor of ABCG2 transporter. SP cells, which disappear in the presence of FTC, were identified by FACS and calculated as a proportion (percentage) of the pool population. The results show that the SP fraction of BSW cells was substantially higher than that of the BC cells (~15% *vs.* 3%), and was comparable to that of the well-established non-small cell lung carcinoma H460 cells, which served as a positive control in this study (Figure 1C). As an additional measure to substantiate the existence of CSCs, we determined CD 133 expression, one of the key biomarkers of lung CSCs [27,28], in BC, BSW, and H460 cells. Figure 1D shows that CD133 expression was high in BSW and H460 cells, but not in BC cells. Altogether, these results supported the notion that BSW cells were enriched with CSCs.

### SP cells display CSC properties

**Figure 2:**
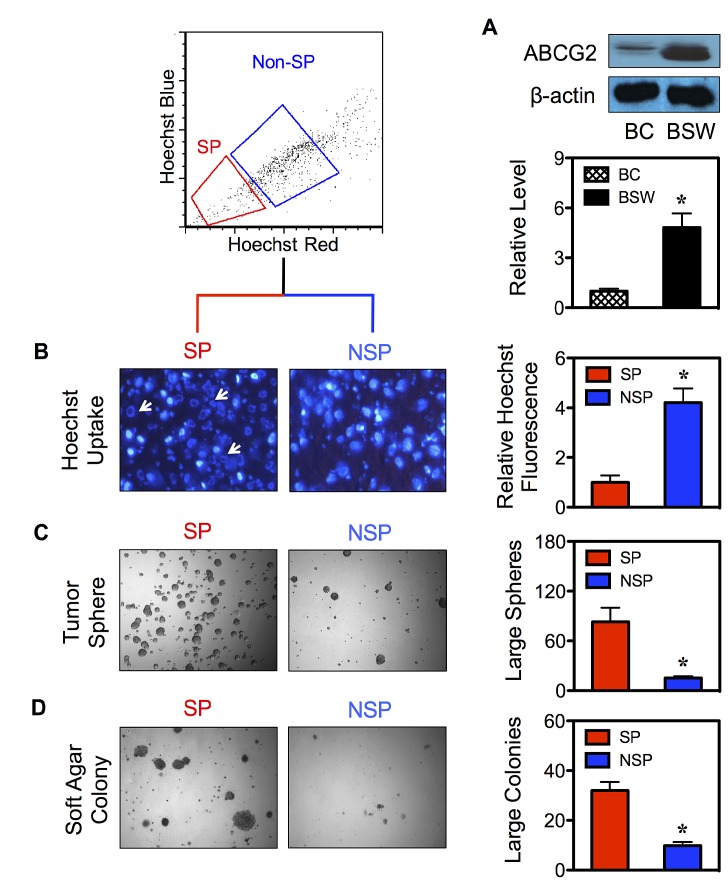
Isolated CSCs display typical CSC characteristics (A) Intrinsic ABCG2 protein expression in control BC and SWCNT-exposed BSW cells was determined by Western blotting. (B) CSCs and their non-CSC counterpart were isolated from BSW cells based on their SP phenotype using FACS and designated as SP and NSP cells, respectively. Fluorescence analysis of Hoechst 33342 blue uptake in SP and NSP cells using fluorescence microscopy (*left*). Arrows indicate the ring-shaped staining pattern in SP cells. Quantitative analysis of the Hoechst blue fluorescence is shown (*right*). (C, D) Analysis of tumor sphere (C) and soft agar colony formation (D) in SP and NSP cells after two weeks of culture.

**Figure 3:**
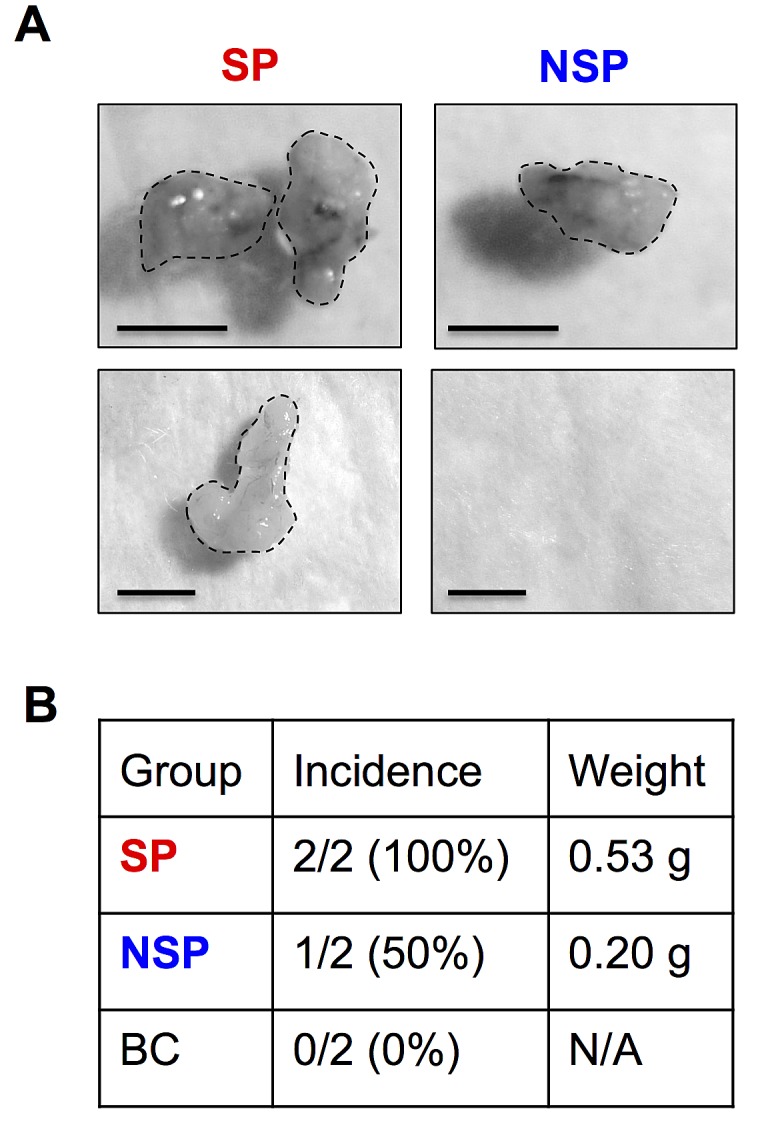
Tumor initiating capability of isolated CSCs Isolated SP and NSP cells derived from chronic SWCNT-exposed BSW cells were subcutaneously injected into the left and right flanks of NSG mice at the injection dose of 5×10^4^cells/flank. (A) SP and NSP tumors at 28 days. (B) Tumor incidence and weight of SP, NSP and BC cells are shown.

FACS enables the isolation of CSCs from their parental cells based on their SP phenotype. To first ensure the basis of SP analysis, we determined the expression level of ABCG2 transporter in BSW cells in comparison with control BC cells. As depicted in Figure 2A, ABCG2 expression was highly upregulated in BSW cells. We then isolated CSCs and their non-CSC counterpart from BSW cells using FACS and designated them as SP and non-SP (NSP) cells, respectively. To validate the stem phenotype of the isolated cells, we assessed their Hoechst dye uptake characteristic using fluorescence microscopy. Figure 2B shows that Hoechst fluorescence intensity was less in the SP compared to NSP cells. We also observed a staining pattern that we called ring-shape pattern in the SP cells (Figure 2B-*arrows*), in which the Hoechst dye cannot completely stain SP nuclei, indicating their ability to efflux the dye. We next subjected freshly isolated SP and NSP cells to tumor sphere and soft agar assays, the gold standards for assessing CSC and malignant properties. The results indicated that the SP cells formed larger and greater number of spheres and colonies than the NSP cells (Figure 2C and D), indicating their CSC features and validity of the CSC isolation. The observation that the NSP cells which accounted for the vast majority of sorted cells but lacked substantial ability to form tumor colonies suggested the role of SP cells (CSCs) as tumor-initiating cells. The greater tumorigenicity of SP cells was subsequently confirmed *in vivo* using a xenograft mouse model, where they exhibited greater tumor incidence, size, and volume (Figure 3A and B).

### SP cells display an aggressive cancer behavior

**Figure 4:**
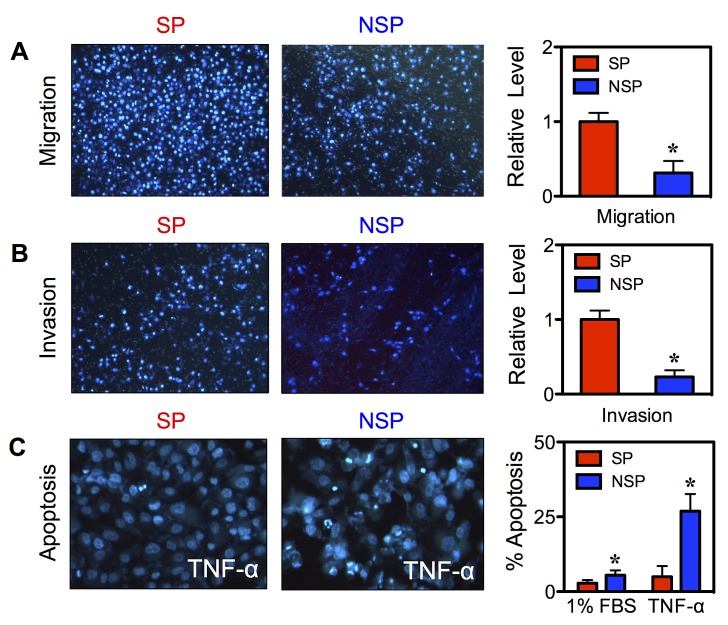
Isolated CSCs display aggressive cancer phenotypes (A, B) Isolated SP and NSP cells derived from chronic SWCNT-exposed BSW cells were analyzed for cell migration (A) and invasion (B) at 48 hours after incubation. (C) Acquired apoptosis resistance of SP cells to TNF-α (150 ng/mL) as compared to NSP cells at 24 hours. Data are mean ± SD (n = 4). *p < 0.05 ***vs.*** SP cells.

The aggressive neoplastic behavior of CSCs was assessed by cell migration, invasion, and apoptosis assays. Freshly isolated SP and NSP cells were seeded onto Transwell chambers with control inserts (migration) or Matrigel-coated inserts (invasion) and incubated for 48 hours. The results showed that the SP cells exhibited a significant increase in migration and invasion activities as compared to NSP cells (Figure 4A and B). This increase in cellular activities was not due to the difference in cell growth since the growth rate of SP and NSP cells was comparable at 48 hours as determined by MTT assay (data not shown). We next compared the apoptosis resistance of SP and NSP cells in response to TNF-α, a known apoptosis inducer of BC cells [12]. Figure 4C shows that TNF-α induced less apoptosis in the SP than NSP cells as demonstrated by their reduced nuclear condensation and fragmentation. These results indicate that CSCs acquired enhanced cell motility and apoptosis resistance, which are important in tumorigenesis and metastasis.

### Gene profiling identifies Cav-1 as an important regulator of tumorigenesis and metastasis

**Figure 5:**
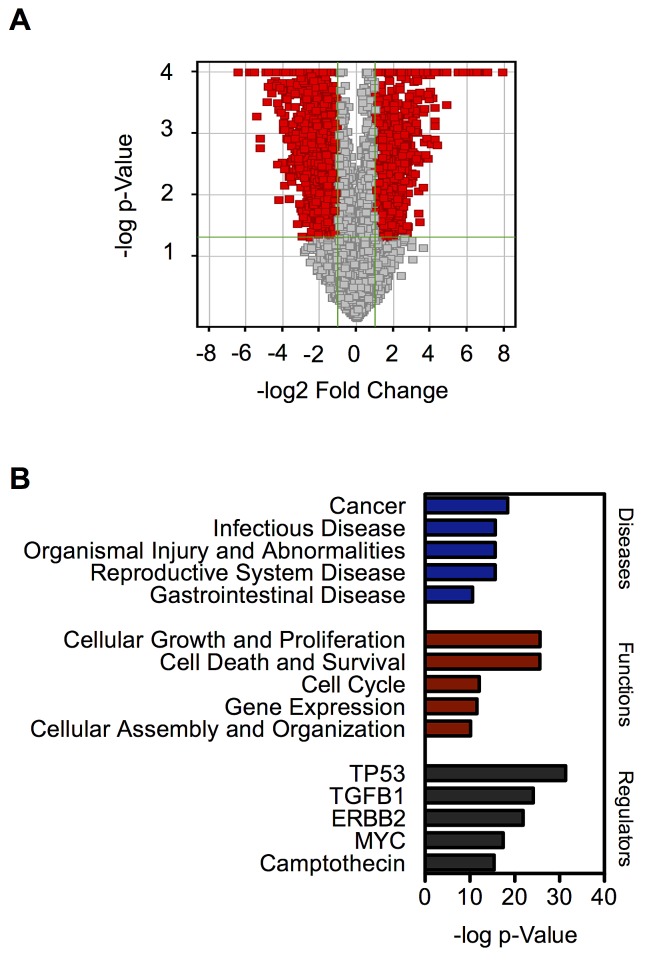
Genome-wide transcription profiles of chronic SWCNT-exposed BSW cells compared to the control BC cells (A) Volcano plot of differentially expressed genes (DEGs) comparing BSW and BC cells. Red point represents DEGs with fold change ≥ 2 and p ≤ 0.05. (B) Gene ontology analysis of top-ranked diseases, functions, and upstream regulators based on p-value.

To gain a better insight into the mechanisms underlying the phenotypic changes of chronic SWCNT-exposed BSW cells, we compared the genome-wide transcription profiles of BSW cells and their passage-control BC cells using microarray analysis. We identified 1932 differentially expressed genes (DEGs) between BSW and BC cells with fold change ≥ 2 and p-value ≤ 0.05, of which 693 genes were upregulated and 1239 genes were downregulated, as shown as red points in the volcano plot (Figure 5A). Gene ontology analysis using Ingenuity Pathway Analysis (IPA; Qiagen, Redwood City, CA) revealed cancer as a top-ranked disease, cell growth/proliferation as a top-ranked cellular function, and *TP53*, the most frequently altered gene in human cancers [29], as a top-ranked upstream regulator (Figure 5B). These results are consistent with our functional assays showing that BSW cells possess aggressive tumorigenic properties.

**Figure 6:**
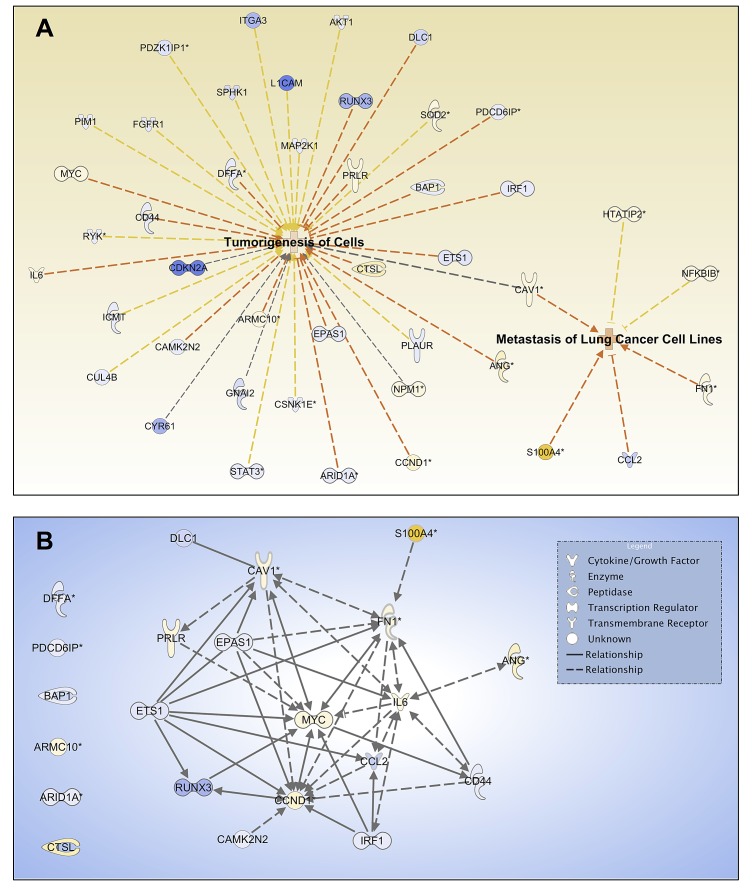
Cav-1 is a potential regulator of tumorigenesis and metastasis (A) Network analysis for the biofunctions of tumorigenesis of cells and metastasis of lung cells based on DEG profiles. Yellow and blue indicate overexpressed and underexpressed genes, respectively, while intensity indicates magnitude of fold change. Orange dash lines indicate predicted activation of the biofunctions based on expression direction. (B) Tumorigenesis and metastasis promoting gene signaling network (GSN). DEGs that promote tumorigenesis of cells and metastasis of lung cells were included in the GSN. Genes were mapped with known direct (solid lines) and indirect (dash lines) signaling associations using IPA. Arrow direction indicates the direction of relationship.

To determine the potential regulator(s) of tumorigenesis and metastasis, which are important features of CSCs, we performed IPA functional analysis of tumorigenesis (p-value 8.74×10^−4^) and metastasis of lung cells (p-value 8.00×10^−4^) based on their DEG profiles. Figure 6A shows that *CAV1*, with an approximately 2.5-fold increase in BSW cells, is a dominant molecule for both networks. Tumorigenesis and metastasis-promoting gene signaling network (GSN) showed that *CAV1* occupied a focal position of the GSN, while other hub genes with first order linkage to *CAV1* include *MYC*, *CCND1, IL6,* and *FN1* (Figure 6B). These findings indicate the importance of *CAV1* in BSW tumorigenesis and metastasis, which may be associated with the CSCs.

**Figure 7:**
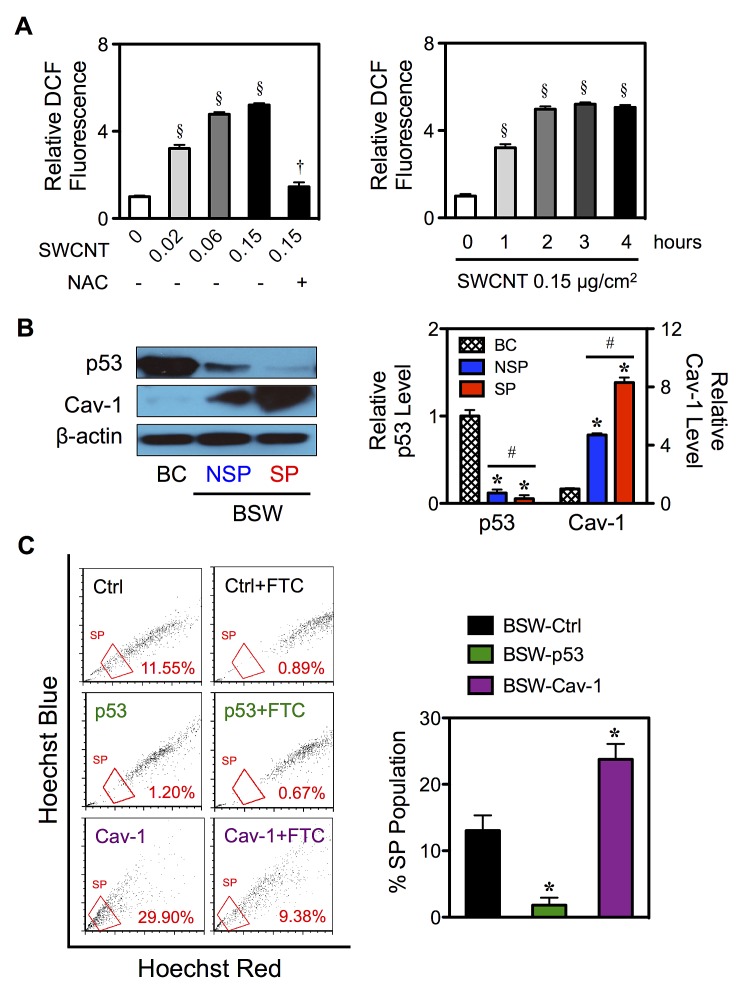
Effects of chronic SWCNT exposure on ROS targeted proteins (A) The effects of SWCNTs on ROS generation of lung epithelial BC cells using H_2_DCF-DA as a fluorescent probe. Cells were treated with varying doses of SWCNTs (0-0.15 μg/cm^2^) in the presence or absence of antioxidant NAC (5 mM) for 3 hours (*left*) or with SWCNTs (0.15 μg/cm^2^) for various times (0-4 hours) (*right*). (B) Isolated SP and NSP cells derived from chronic SWCNT-exposed BSW cells were analyzed for tumor suppressor p53 and tumor promoter Cav-1 protein expression using Western blotting in comparison with their passage-matched control BC cells. β-actin was used as a loading control. The intensity of p53 and Cav-1 bands was normalized to that of β-actin. Relative intensity level was then calculated by dividing the normalized intensity of each sample by that of the control, i.e. in BC cells. (C) BSW cells were stably transfected with empty vector (Ctrl), p53 and Cav-1 plasmids as described in Materials and methods. Analysis of side population (SP) in BSW-Ctrl, BSW-p53 and BSW-Cav-1 cells using FACS. SP cells (box) were determined by their disappearance in the presence of FTC and were plotted as a percentage of pool population. Data are mean ± SD (n = 3). ^§^p < 0.05 ***vs.*** untreated BC cells. ^†^p < 0.05 *vs*. SWCNT-treated BC cells (0.15 μg/cm^2^). *p < 0.05 *vs.* vector-transfected BSW-Ctrl cells. #p < 0.05 *vs*. SP cells.

Oxidative stress, the second top-ranked toxicological responses on the IPA (p-value 2.53×10^−5^), has been shown to be induced in the tumor microenvironment [30,31] and has been suggested to play a vital role in tumorigenesis and metastasis [32,33]. Here we show that treatment of the cells with SWCNTs (0-0.15 μg/cm^2^) induced a dose- and time-dependent increase in cellular DCF fluorescence, an indicator of cellular ROS generation and oxidative stress, in BC cells (Figure 7A). An addition of antioxidant *N*-acetylcysteine (NAC, 5mM) inhibited the oxidative effect of SWCNTs (0.15 μg/cm^2^) (Figure 7A-*left*), thus confirming the induction of ROS by SWCNTs.

### Role of p53 and Cav-1 in CSC induction

To understand the underlying mechanisms of CSC induction by SWCNTs, we investigated the potential role of p53 and Cav-1 since they are known regulators of lung carcinogenesis [34,35] and are key targets of ROS [21,22,36]. Their potential role in BSW tumorigenesis was also suggested by IPA. Figure 7B shows that p53 was strikingly downregulated in SP cells with the rank order of expression being BC > NSP > SP. In contrast, Cav-1 expression was highest in the SP cells followed by NSP and BC cells (SP > NSP > BC). To further investigate the functional role of p53 and Cav-1 in CSC induction, we manipulated their expression by gene transfection in chronic SWCNT-exposed BSW cells and studied their effect on SP phenotype. Stably transfected cells were stained with Hoechst 33342 in the presence or absence of ABCG2 inhibitor FTC, and SP fractions were determined as earlier described. Figure 7C shows that overexpression of p53 significantly inhibited SP, whereas overexpression of Cav-1 promoted SP as compared to parental BSW cells. These results indicate the involvement of p53 and Cav-1 in the CSC acquisition of SWCNT-treated cells.

**Figure 8:**
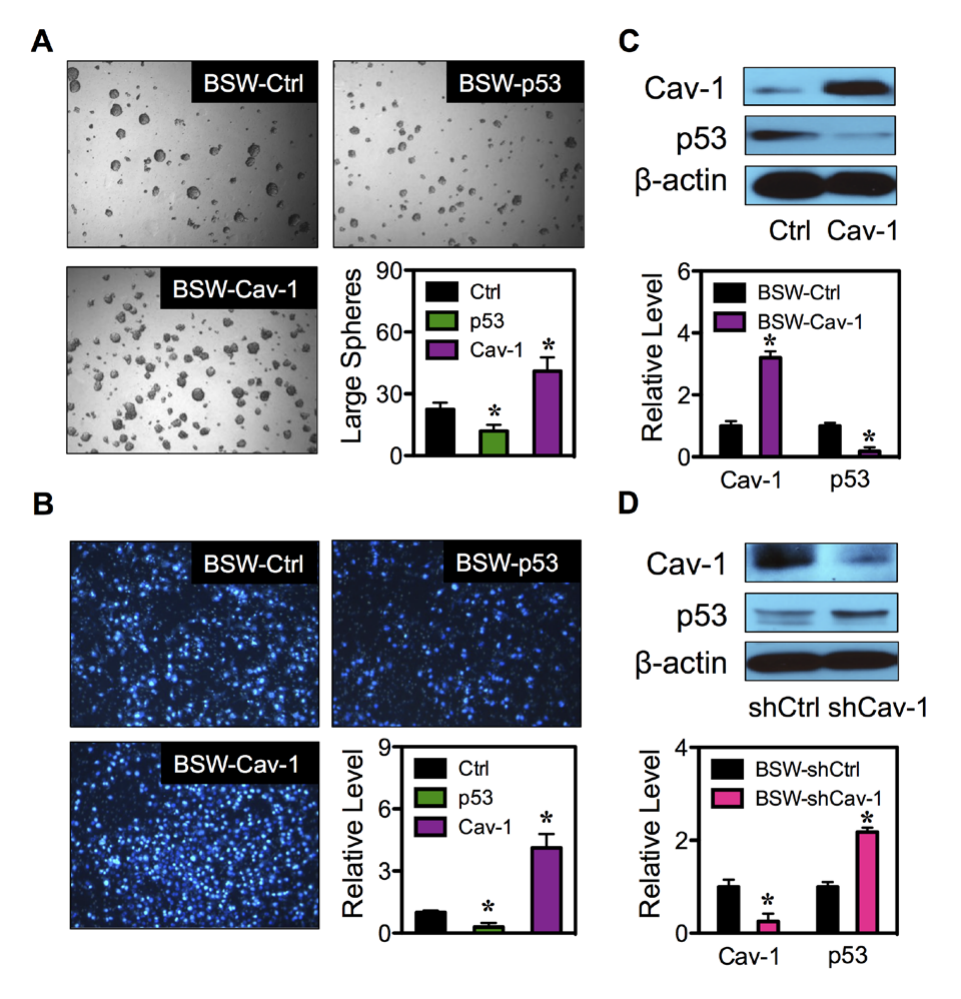
Cav-1 regulates p53 expression (A) Analysis of tumor spheres in BSW-Ctrl, BSW-p53 and BSW-Cav-1 cells after two weeks of culture. (B) Cell migration assays of BSW-Ctrl, BSW-p53 and BSW-Cav-1 cells at 48 hours after incubation. (C) Effect of Cav-1 overexpression on p53 expression. Cav-1 and p53 expression in BSW-Ctrl and BSW-Cav-1 cells were determined by Western blotting. β-actin was used as a loading control. (D) Cav-1 knockdown experiments were performed using Cav-1 shRNA (shCav-1) and Cav-1 and p53 expression in BSW-shCtrl (vector) and BSW-shCav-1 cells were analyzed by Western blotting. Data are mean ± SD (n = 3). *p < 0.05 *vs*. vector-transfected BSW-Ctrl or BSW-shCtrl cells.

To confirm the role of p53 and Cav-1 in CSC regulation, tumor sphere formation assays were performed on the genetically manipulated cells. Figure 8A shows that the p53-overexpressing cells formed smaller and fewer number of tumor spheres, while the Cav-1-overexpressing cells exhibited larger and greater number of tumor spheres as compared to vector-transfected BSW control cells, thus validating the inhibitory role of p53 and the promoting role of Cav-1 in the CSC induction by SWCNTs.

### Role of p53 and Cav-1 in cell aggressiveness

Having shown that the CSCs acquired aggressive cancer phenotypes, we determined whether overexpression of p53 and Cav-1 affect cell aggressiveness by analyzing cell migration using Transwell migration assay. Figure 8B shows that cell migration was substantially reduced in p53-overexpressing cells as compared to vector-transfected control cells, while Cav-1-overexpressing cells exhibited increased migratory activity. These results indicate the role of p53 and Cav-1 in cell aggressiveness and strengthen their role in CSC regulation.

### Regulation of p53 by Cav-1 in SWCNT-treated cells

Previous studies showed that an increased expression of Cav-1 abrogated p53 activation in breast cancer cells induced by cell detachment through an up-regulation of insulin-like growth factor I (IGF-I) receptor [37]. In this study, we similarly observed the inverse correlation between Cav-1 and p53 in SP and NSP cells (Figure 7B). To test the potential regulatory axis of the two molecular regulators in controlling CSC induction, we genetically altered Cav-1 expression in BSW cells by stable gene transfection and studied its effect on p53 expression. Figure 8C shows that an overexpression of Cav-1 resulted in a decrease in p53 expression as compared to control-transfected cells. To confirm the above finding, gene knockdown experiments were conducted in BSW cells. The cells were transfected with Cav-1 shRNA viral particles and analyzed for Cav-1 and p53 expression. Figure 8D shows that as compared to control-transfected cells, the shCav-1-transfected cells exhibited substantially higher p53 expression. Together, these results indicate Cav-1 as a negative regulator of p53 in BSW cells.

### Cav-1 promotes tumor growth and progression *in vivo*

**Figure 9:**
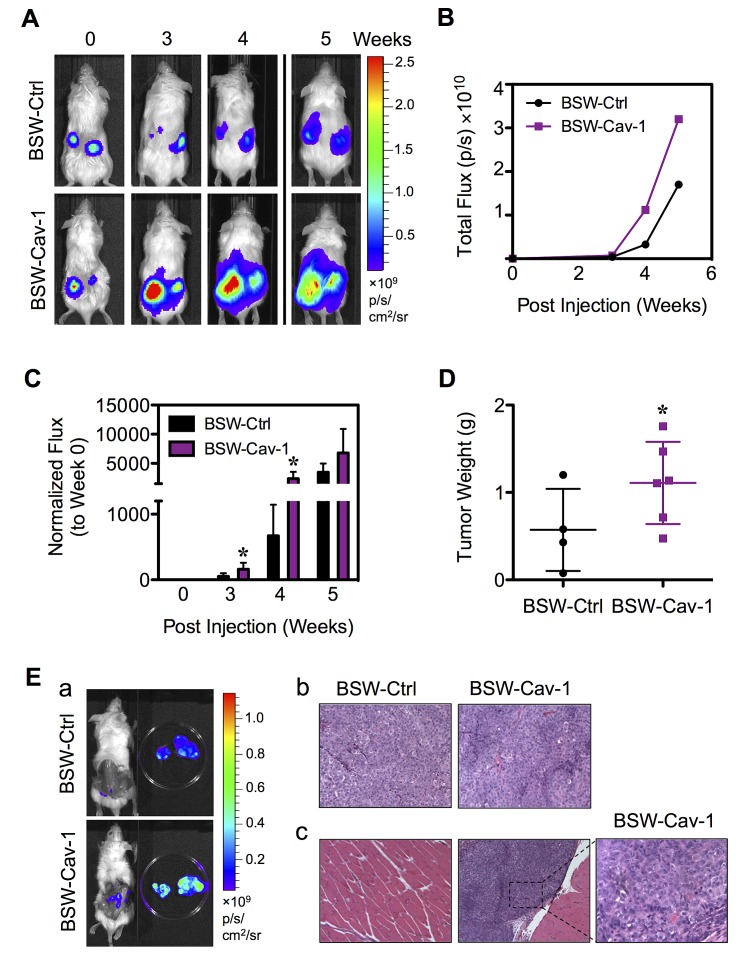
Cav-1 promotes tumor growth and progression BSW-Ctrl and BSW-Cav-1 cells were genetically labeled with luciferase and subcutaneously injected into the left and right flanks of NSG mice at the injection dose of 3×10^5^/flank. (A) Representative sequences of bioluminescence images of mice from BSW-Ctrl and BSW-Cav-1 cells taken at the time of inoculation (week 0) and 3-5 weeks post-injection. (B) Quantitative analysis of tumor bioluminescence signals over time. (C) Normalization of tumor signals at various times to their initial signal at week 0. (D) Subcutaneous tumors were dissected and weighed at 5 weeks post-injection. (E) (a) Representative sequences of bioluminescence images of mice and dissected subcutaneous tumors. (b, c) Representative H&E micrographs of subcutaneous tumors (b) and neighboring muscle tissues (c). Data are mean ± SD (n = 4 or 6). *p< 0.05 ***vs.*** vector-transfected BSW-Ctrl cells.

To confirm the role of Cav-1 in the tumorigenicity of BSW cells, we assessed tumor growth and progression *in vivo* using a xenograft mouse model. To link Cav-1 to CSC regulation, we transfected the parental BSW cells, which express a low level of Cav-1 relative to their CSC subpopulation, with Cav-1 or control plasmid. Cav-1-overexpressing and control-transfected cells were then genetically labeled with luciferase (Capital Biosciences, Rockville, MD) and subcutaneously injected into NSG mice at 3×10^5^ cells/flank. Injection of the control BC cells at the same (3×10^5^ cells) or higher (1×10^6^ cells) doses did not induce tumor formation in NSG mice (data not shown). Tumor growth was monitored weekly by using IVIS^®^ bioluminescence imaging. By three weeks post-injection, the tumor luminescence was strikingly higher in mice bearing Cav-1-overexpressing cells as compared to control cells (Figure 9A), indicating the positive regulatory role of Cav-1 on tumor growth. Quantitative analysis of the tumor luminescence signals over time was shown in Figure 9B. For comparison, these signals were normalized to their initial signals at the time of inoculation (week 0). Figure 9C shows that the tumor signals were significantly higher in mice bearing Cav-1-overexpressing cells at 3 and 4 weeks post-injection. The maximum of approximately 4-fold tumor induction was observed with Cav-1-overexpressing cells over control cells, substantiating the tumor promoting role of Cav-1. At the end of the experiment (week 5), subcutaneous tumors were dissected and tumor weight was compared between groups. The greater weight of Cav-1-overexpressing tumors further supported Cav-1 as a positive regulator of tumorigenesis (Figure 9D). Histological analysis of subcutaneous Cav-1-overexpressing tumors by H&E staining showed a classical cancer cell morphology including the condensation of heterochromatin and the presence of multinucleated cells, an indicator of mitotic dysfunction (Figure 9E-b).

Interestingly, we observed a remarkable tumor bioluminescence in mice bearing Cav-1-overexpressing cells after the dissection of subcutaneous tumors (Figure 9E-a). Histological analysis of neighboring muscle tissues showed metastatic cells in the muscle tissues of mice bearing Cav-1-overexpressing cells (Figure 9E-c). This result indicates metastasis of Cav-1-overpressing cells to neighboring tissues, which is consistent with the *in vitro* result showing the increased aggressiveness of cells by Cav-1 (Figure 8B), and suggests an important role of Cav-1 in tumor metastasis.

## DISCUSSION

This study addresses human lung cancer risks associated with chronic pulmonary exposure to CNTs. We report here that chronic exposure of non-tumorigenic human lung epithelial cells to SWCNTs, a major form of engineered CNTs, can directly initiate the formation of lung CSCs, as indicated by tumor sphere and SP assays (Figure 1). The acquisition of these CSCs is important for their malignant and aggressive properties. This conclusion is based on the observations that the isolated CSCs (SP cells) formed much larger colonies and spheres under soft agar and tumor sphere assays (Figure 2), had greater tumor-initiating capability *in vivo* (Figure 3), and possessed increased migratory and invasive activities as well as apoptosis resistance phenotype as compared to non-CSCs (NSP cells) (Figure 4). Consistent with this finding, a recent study similarly reported the acquisition of stem-like properties by lung epithelial cells chronically exposed to tobacco and arsenic carcinogens [38,39] with enhanced cell motility [38]. Thus, these results may reflect common cellular responses to chronic carcinogen exposure.

ROS are known to play a key role in tumor development as they may cause damage to DNA, RNA and proteins, leading to changes in chromosome instability, gene mutation and altered gene expression [32]. SWCNTs induced a significant level of ROS, the effect of which inhibited by antioxidant NAC (Figure 7A). In response to chronic SWCNT exposure, we observed the striking alterations of tumor regulators and ROS targeted proteins, including downregulation of tumor suppressor p53 and upregulation of tumor promoter Cav-1 (Figure 7B), consistent with their suggested role by IPA gene ontology analysis. Using gene manipulation, we revealed for the first time the involvement of p53 and Cav-1 in CSC acquisition and cell aggressiveness induced by SWCNTs (Figure 7C, 8A and B). It has been recently demonstrated that wild-type p53 positively regulated cell differentiation in breast cancer cells and loss of p53 favored CSC growth [40,41]. However, the functional role of Cav-1 in CSC induction is not known. Our study provides the first initial evidence for the tumor promoting activity of Cav-1 through CSC regulation. Furthermore, we found that Cav-1 acts upstream of p53 in mediating the effect (Figure 8C and D). Overexpression of Cav-1 leads to p53 inactivation in the tested cell system, consistent with the earlier finding in breast cancer cells [35]. By contrast, p53 was activated upon Cav-1 inhibition by shRNA knockdown. In addition to the regulatory role of ROS in Cav-1 expression [22], the feedback antioxidant function of Cav-1 was reported in human lung cancer cells [42]. As p53 activation is generally triggered by ROS [34], it is plausible that the observed p53 inactivation may result from the antioxidant effect of Cav-1. Cav-1 and p53 may form a regulatory axis that regulates CSCs through c-Myc, one of the original stem cell reprogramming factors [43,44] and a proto-oncogene [45]. Tumorigenesis and metastasis promoting GSN revealed *MYC* overexpression and its first order linkage to *CAV1* (Figure 6B). Cav-1 overexpression and inhibition have previously shown to mediate c-Myc level in prostate cancer cell lines through the interaction between Cav-1 and low-density lipoprotein receptor-related protein 6 (LRP6) [46]. Likewise, c-Myc is a known downstream target of p53 at the transcriptional and posttranscriptional levels [47,48]. With its unique structure of lipid microdomains, Cav-1 interacts with multidrug resistance ABCG2 transporter protein and regulates its transporting activity [49,50]. As SP phenotype of CSCs is attributed to the expression of ABCG2, it is plausible that Cav-1 might also regulate CSCs through ABCG2 protein.

Having demonstrated that Cav-1 contributed to CSC regulation and malignancy induced by chronic SWCNT exposure *in vitro*, we determined the role of Cav-1 in tumor growth and progression *in vivo*. Our data showed that Cav-1 not only promoted tumor formation but also metastasis of the tumor cells to neighboring tissues (Figure 9). The tumor-promoting effect of Cav-1 in SWCNT-transformed cells is similar to that observed in human lung cancer cell lines [51], indicating a potential shared mechanism between laboratory SWCNT-driven tumors and human lung cancer.

In conclusion, this study demonstrated that SWCNTs are capable of inducing CSCs and tumorigenesis in a xenograft mouse model. This study also unveiled the positive regulatory role of Cav-1 in the tumorigenic process. This protein is overexpressed in chronic SWCNT-exposed lung cells, particularly in the CSC subpopulation, and is a key contributing factor to the malignant and aggressive properties of the cells. Cav-1 acts upstream of p53 resulting in its inactivation, the event that is common in most human cancers [27]. By linking Cav-1, CSCs and tumorigenesis, we documented a novel mechanism of Cav-1 that drives SWCNT tumorigenesis possibly through CSC acquisition. As induction of CSCs is an early event in carcinogen-induced pre-malignancy [38], detection of Cav-1 might aid in the early diagnosis and prevention of CNT-induced lung carcinogenesis.

## MATERIALS AND METHODS

### CNT characterization and preparation

SWCNTs were obtained from Carbon Nanotechnology (CNI, Houston, Texas) and were purified by acid treatment to remove metal contaminates. Elemental analysis by nitric acid dissolution and inductive coupled plasma-atomic emission spectrometry (ICP- AES) showed that the purified SWCNT contained 99% elemental carbon and less than 1% of contaminants. The metal residues were mostly iron (Fe) at 0.23% by weight. The surface area, length, and width of individual dry SWCNT were 400-1040 m^2^/g, 0.6 ± 0.5 μm, and 1 ± 0.2 nm (W), respectively [52]. SWCNTs were dispersed by using acetone/sonication method as previously described [12].

### Chemicals and reagents

Hoechst 33342, ***N***-acetyl cysteine (NAC), dihydrodichlorofluorescein diacetate (H_2_DCF-DA), and antibody against β-actin were obtained from Sigma-Aldrich (St. Louis, MO). TNF-α was obtained from EMD Biosciences (La Jolla, CA). Antibody against Cav-1 and p53 was obtained from Santa Cruz Biotechnology (Santa Cruz, CA). Antibodies against peroxidase-labeled secondary antibody were obtained from Cell Signaling Technology (Boston, MA).

### Cell culture

Human bronchial epithelial BEAS-2B cells were obtained from American Type Culture Collection (ATCC; Manassas, VA). Cells were cultured in Dulbecco's modified Eagle medium (DMEM) supplemented with 5% fetal bovine serum (FBS), 2 mM L-glutamine, 100 units/mL penicillin and 100 μg/mL streptomycin (Gibco, Gaithersburg, MA). Non-small cell lung cancer H460 cells were obtained from ATCC and were cultured in RPMI 1640 medium supplemented with 5% FBS, 2 mM L-glutamine, and 100 units/mL penicillin/streptomycin. Both cells were maintained in a humidified atmosphere of 5% CO_2_ at 37 °C.

### Plasmid and transfection

Cav-1, p53, and control GFP plasmids were obtained from Invitrogen (Carlsbad, CA). Cells were transfected with Cav-1, p53, or GFP plasmid by nucleofection using Nucleofector^®^ (Amexa Biosystems, Cologne, Germany), according to the manufacturer's instructions. Briefly, cells were suspended in 100 μL of nucleofection solution with 2 μg of plasmid and nucleofected using the device program T020. The cells were then resuspended in 500 μL of complete medium and seeded in 60-mm cell culture dishes. Cells were allowed to recover for 48 hours before each experiment and were cultured for 28 days in G418-containing medium (800 μg/mL). The pool stable transfectant was identified by Western blotting of Cav-1 and p53 and was cultured in the G418-free medium for at least three passages before each experiment.

### Inhibition of Cav-1 by RNA interference

Lentiviral transduction particles carrying short hairpin RNA (shRNA) sequence against human Cav-1 (5'-CCGGGACGTGGTCAAGATTGACTTTCTCGAGAAAGTCAATCTTGACCACGTCTTTTT-3') and control nontarget sequence (5'-CCGGCAACAAGATGAAGAGCACCAACTCGAGTTGGTGCTCTTCATCTTGTTGTTTTT-3') were used to knockdown Cav-1 expression in BSW cells. The viral vectors were obtained commercially from Sigma (accession numbers NM_001753 and SHC002V) and were used according to the manufacturer's instruction. Briefly, cells were seeded in 6-well plates (5×10^5^/well) and incubated with Cav-1 shRNA lentiviral particles at the multiplicity of infection of 1.5 in the presence of hexadimethrine bromide (8 μg/mL) for 36 hours. Transfected cells were analyzed for Cav-1 by Western blotting prior to use.

### Chronic CNT exposure and derivation of CNT-transformed cells

Non-tumorigenic human lung epithelial BEAS-2B cells were continuously exposed to non-cytotoxic SWCNTs at 0.02 μg/cm^2^ in culture for 6 months. This dose, which is equivalent to a human lung burden for 8 hours/day over a month at 400 μg/m^3^ (high CNT level reported in a research facility) [53] or about 3 years at 10 μg/m^3^ (average CNT level in U.S. facilities) [54], was calculated from the lowest dose which induced positive *in vivo* biological response (10 μg/mouse lung). The cells were passaged weekly at preconfluent densities using a solution containing 0.05% trypsin and 0.5 mM EDTA (Invitrogen, Carlsbad, CA). SWCNT-exposed BEAS-2B cells were designated as BSW cells. Parallel cultures grown in CNT-free medium with the same background level of dispersant provided passage-matched control and were designated as BC cells. After 6 months of exposure, the cells were cultured in normal complete medium, and their cancer and CSC phenotypes were assessed as described below.

### Soft agar colony formation assay

Passage-control BC and chronic SWCNT-exposed BSW cells (3×10^4^ cells) were mixed with culture medium containing 0.5% agar to a final agar concentration of 0.33%. Cell suspensions were immediately plated onto dishes coated with 0.5% agar in culture medium. Colonies were examined under a light microscope after 2 weeks of culture. In order to assess the self-renewing property of cells, spheres were collected by gentle centrifugation, dissociated into single cell suspensions, filtered and cultured under conditions described above (second spheres).

### Tumor sphere assay

Tumor sphere assay was performed under non-adherent and serum-free conditions as previously described as stem cell-selective conditions [24,25]. Briefly, cells were resuspended in 0.8% methylcellulose (MC)-based serum-free medium (Stem Cell Technologies, Vancouver, Canada) supplemented with 20 ng/mL epidermal growth factor (BD Biosciences, San Jose, CA), basic fibroblast growth factor and 4 mg/mL insulin (Sigma) and plated at 5×10^3^ cells. Cells were then cultured for two weeks.

### Side population analysis and isolation

Cells were detached by trypsinization and 1×10^6^ cells were labeled with 5 μg/mL of Hoechst 33342 in DMEM-F12 medium containing 2% FBS in the presence or absence of 25 μM ABCG2 inhibitor fumitremorgin C (FTC; EMD Biosciences, San Diego, CA) at 37 °C for 90 minutes. The cells were then centrifuged and resuspended in ice-cold Hank's buffer salt solution (HBSS). Side population (SP) analysis and sorting were performed using BD FACSAria fluorescence-activating (flow cytometry)-based cell sorter (FACS; BD Biosciences). The Hoechst dye was excited with a UV laser and its fluorescence was measured with both a 450/20 filter (Hoechst Blue) and 675 LP filter (Hoechst Red). SP fraction was calculated based on the disappearance of SP cells in the presence of FTC using the formula: SP percentage in the absence of FTC − SP percentage in the presence of FTC.

### Hoechst uptake

Cells were directly stained with 5 μg/mL of Hoechst 33342 in phosphate-buffered saline (PBS) in the culture plates at 37°C for 30 minutes. Hoechst 33342 blue uptake was analyzed by fluorescence microscope using a 350-nm excitation and 450-nm emission beam.

### Cell migration and invasion assays

*In vitro* cell migration and invasion were determined using a 24-well Transwell^®^ unit with polycarbonate (PVDF) filters (8-μm pore size). The membrane was coated with Matrigel^®^ (BD Biosciences, NJ) for the invasion assay, while control inserts were used for the migration assay. Briefly, cells at the density of 3×10^4^ cells per well (invasion) or 1.5×10^4^ cells per well (migration) were seeded into the upper chamber of the Transwell^®^ unit in serum-free medium. The lower chamber of the unit was filled with a normal growth medium containing 5% FBS. Chambers were incubated at 37 °C in a 5% CO_2_ atmosphere for 48 hours. The non-migrating or non-invading cells were removed from the inside of the insert with a cotton swab. Cells that migrated or invaded to the underside of the membrane were fixed and stained with 10 μg/mL Hoechst 33342 for 30 minutes. Inserts were visualized and scored under a fluorescence microscope (Leica DM, IL). Cell migration and invasion was expressed as the ratio of migrated or invaded cells from the treated and control sample (relative level).

### Apoptosis assay

Apoptosis was determined by DNA condensation/fragmentation assay using Hoechst 33342 dye. Cells were incubated with 10 μg/mL of Hoechst 33342 for 30 minutes and visualized under a fluorescence microscope (Leica Microsystems, Bannockburn, IL). Cells having intensely condensed and/or fragmented nuclei were considered apoptotic. Approximately 1000 nuclei from 10 random fields were analyzed for each sample. The apoptotic index was calculated as the percentage of cells with apoptotic nuclei over total number of cells.

### Whole genome expression microarray and gene ontology analysis

Whole genome expression was determined using high-throughput mRNA microarray analysis following MIAME guidelines as described previously [48]. Briefly, cells (1×10^6^ in 6-cm plate) were lysed in triplicate using TRIzol^®^ reagent (Life Technologies, Grand Island, NY), snap frozen in liquid nitrogen and shipped overnight to ArrayStar (Rockville, MD). Total RNA was isolated, purified and quantified using Nanodrop ND-1000 (Thermo Scientific, Rockford, IL). RNA was tested for purity and DNA contamination using A260/A280 ration and standard denaturing agarose gel electrophoresis. Gene expression profiling was performed using NimbleGen Human 12×135k Gene Expression Array using the NimbleGen Hybridization System (Roche NimbleGen, Madison, WI). The slides were scanned with Axon GenePix 4000B microarray scanner (Molecular Devices Corporation, Sunnyvale, CA). Raw data intensities were extracted from the aligned scanned images and normalized through quantile normalization and Robust Multichip Average method in NimbleGen v2.5. Gene level files were imported into Agilent GeneSpring GX (v12.1) for analysis. Genes with < 50.0 intensity were removed from further analysis. Volcano plots were constructed using two sample t-tests (p ≤ 0.05) and ± 2-fold change filter criteria to identify differentially expressed genes (DEGs) in chronic SWCNT-exposed BSW cells compare to control BC cells. All gene expression data were deposited to NCBI's Gene Expression Omnibus and is accessible via accession number (GenBank ID: GSE56104). Gene ontology, gene network, and upstream prediction analysis was performed using Ingenuity Pathway Analysis (IPA; Qiagen, Redwood City, CA).

### ROS detection

ROS generation was determined fluorometrically using H_2_DCF-DA as a fluorescent probe. Briefly, cells were incubated with the probe (10 μM) for 30 min at 37°C, after which they were washed and resuspended in PBS, followed by analysis of cellular fluorescence intensity by a fluorescence plate reader at the excitation and emission wavelengths of 485 and 538 nm, respectively.

### Western blot analysis

After specific treatments, cells were incubated in lysis buffer containing 20 mM Tris-HCl (pH 7.5), 1% Triton X-100, 150 mM NaCl, 10% glycerol, 1 mM Na_3_VO_4_, 50 mM NaF, 100 mM phenylmethylsulfonyl fluoride, and a commercial protease inhibitor mixture (Roche Molecular Biochemicals, Indianapolis, IN) at 4 °C for 20 minutes. The lysate was collected and determined for protein content using the Bradford method (Bio-Rad Laboratories, Hercules, CA). Proteins (40 μg) were resolved under denaturing conditions by 7.5-12% sodium dodecyl sulfate-polyacrylamide gel electrophoresis (SDS-PAGE) and transferred onto nitrocellulose membranes (Bio-Rad). The transferred membranes were blocked for 1 hour in 5% nonfat dry milk in TBST (25 mM Tris-HCl, pH 7.4, 125 mM NaCl, 0.05% Tween 20) and incubated with the appropriate primary antibodies at 4 °C overnight. Membranes were washed twice with TBST for 10 minutes and incubated with HRP-coupled isotype-specific secondary antibodies for 1 hour at room temperature. The immune complexes were detected by an enhanced chemiluminescence detection system and quantified using analyst/PC densitometry software.

### Xenograft mouse model

Animal care and experimental procedures described in this study were performed in accordance with the Guidelines for Animal Experiments at West Virginia University with the approval of the Institutional Animal Care and Use Committee (IACUC #12-0502). Immunodeficient NOD/SCID gamma mice, strain NOD.Cg-Prkdc^scid^ Il2rg^tm1Wjl^/SzJ (NSG; Jackson Laboratory, Bar Harbor, ME), were maintained under pathogen-free conditions within the institutional animal facility. Food and tap water were given ad libitum. Mice were subcutaneously injected with either 5×10^4^ sorted SP/NSP cells or 3×10^5^ luciferase-labeled cells suspended in 100 μL of ExtraCel^®^ hydrogel (Advanced BioMatrix, San Diego, CA). Mice were inspected daily for any signs of distress such as weight loss, hunching, failure to groom, and red discharge from the eyes. Tumor growth of luciferase-labeled cells was monitored weekly by using *in vivo* IVIS^®^ imaging. At the end of experiments, mice were euthanized and tumors were dissected and weighted. Tumor specimens were cut into 5-μm sections and stained with hematoxylin and eosin (H&E) to confirm the cancer-specific morphology and cellular structure. Tumor metastasis to neighbor tissues was analyzed by IVIS^®^ imaging after removal of primary SC tumors. All tissue sectioning and staining were performed at the West Virginia University Pathology Laboratory for Translational Medicine.

### Statistical analysis

The data represent means ± SD from three or more independent experiments as indicated. Statistical analysis was performed by Student's t test at a significance level of p < 0.05.

### FUNDING

This work was supported by the National Institute for Occupational Safety and Health and by grants from National Institutes of Health [R01-HL095579and R01-ES022968 to Y.R.]; National Science Foundation [EPS-1003907 to Y.R.], and Mary Babb Randolph Cancer Center Sara C. Allen Lung and James F. Allen Comp Lung Cancer Research Fund [to S.L.]. Flow cytometric analysis was performed in the West Virginia University Flow Cytometry Core Facility, which is supported in part by National Institutes of Health [P30-GM103488]. Microarray data analysis was performed in support of West Virginia Idea Network of Biomedical Research Excellence (WV-INBRE) [GM103434]. Animal experiments were performed in the West Virginia University Animal Models and Imaging Facility, which is supported in part by the Mary Babb Randolph Cancer Center and National Institutes of Health Grants [P20-RR016440, P30-RR032138/GM102488, S10-RR026378].
